# A systematic review of randomized control trials looking at functional improvement of rotator cuff partial thickness tears following platelet-rich-plasma injection: a comparison of glenohumeral joint vs. subacromial bursa vs. intratendinous injection locations

**DOI:** 10.1016/j.jseint.2024.01.003

**Published:** 2024-01-24

**Authors:** Michael Dakkak, Arhum Saleem, Dev Patel, Matthew Yeager, Leonardo Oliveira, Gregory Gilot, Kurt P. Spindler

**Affiliations:** a Levitetz Department of Orthopaedic Surgery, Cleveland Clinic Florida, Weston, FL, USA; b Herbert Wertheim College of Medicine, Florida International University, Miami, FL, USA

**Keywords:** PRP, Partial thickness rotator cuff tears, Intra-articular injection, Subacromial injection, Intratendinous injection, Corticosteroid

## Abstract

**Background:**

Prior research has demonstrated that platelet-rich plasma (PRP) has shown promising results in the treatment of knee osteoarthritis, lateral epicondylitis, and rotator cuff disease. However, there is a lack of standardization with PRP regarding its use for partial thickness rotator cuff tears (PTRCTs). The primary objective of this review is to assess the location of PRP injections in the shoulder, and how it corresponds to shoulder functional outcomes in PTRCTs.

**Methods:**

Data sources included randomized controlled trials (RCTs) conducted between January 2010 and September 2021 with the terms PRP, partial thickness rotator cuff tears, intra-articular injections, subacromial injections, and intratendinous injections. Major inclusion criteria: partial thickness rotator cuff tears only, functional outcome scores pre-injection and post-injection, minimum 2-month follow-up time, and nonsurgical PRP injections only. Major exclusion criteria: PRP used as an adjunct therapy, full-thickness rotator cuff tears, and surgical intervention before treatment.

**Results:**

A total of 8 RCTs were included which utilized PRP injected into the shoulder for PTRCTs. Studies were grouped by the location of the injection with the following breakdown: 1 glenohumeral joint, 4 subacromial bursa, and 3 intratendinous as the site of injection of PRP. Intra-articular PRP showed a 46.2% improvement (*P* < .05) in the Disabilities of the Arm, Shoulder, and Hand score at 12-month follow-up, however PRP compared to physical therapy had no statistical difference. For subacromial injections, one study showed no statistical difference between hyaluronic acid and PRP vs PRP, but both groups showed improvement compared to normal saline at 3, 6, and 12 months (*P* < .05). For intratendinous injections, PRP was found to be superior in the Shoulder Pain and Disability Index scores at 66.1% improvement (*P* < .05) at 3 months and 71.6% at 6 months (*P* < .05) after two PRP injections when compared to dry needling. Another study showed a statistically significant difference in ASES score when combining LP-PRP injection intratendinous and subacromial bursa when compared to corticosteroid at 3 months. Furthermore, at 6-month follow-up, the PRP group showed significant improvement in the Oxford Shoulder Score compared to a subacromial bursa corticosteroid group 53.8% vs 31.7% (*P* < .01).

**Conclusion:**

Based on our review of current literature, there is inconclusive evidence of the ideal location to inject PRP when partial rotator cuff tear is present. Despite PRP showing improved functional outcomes in patients diagnosed with PTRCT regardless of the injection site, more research is needed to figure out the optimal concentration of PRP, frequency of injection, and who are ideal candidates when utilizing PRP for PTRCTs.

Partial thickness rotator cuff tears (PTRCTs) are a common shoulder pathology seen with its prevalence increasing with age with roughly 4% at age < 40 years and 26% at age > 60 years.^14^^,^^23^^,^^25^ The pathophysiology of PTRCTs is usually multifactorial, consisting of intrinsic and extrinsic factors. These factors can be age-related cellular changes leading to microtears, instability, decreased vascularity, smoking, hand dominance, and postural adaptations.^17^ Repeated mechanical burden results in stress to the tenocytes and extracellular matrix leading to collagen remodeling and ultimately apoptosis which weakens the tendon and can lead to tearing.^8^

From a clinical perspective, it is important to diagnose and treat partial rotator cuff tears (PRCTs) before they progress to full-thickness rotator cuff tears and cause functional impairment. The rate of progression for PTRCTs into full-thickness tears is scattered throughout the literature, with up to 40% of symptomatic PTRCTs developing into full-thickness tears.^15^ Pain development in shoulders with an asymptomatic rotator cuff tear is associated with an increase in tear size.^4^ The progression rate of a PRCT of > 5mm difference over 6 months with follow-up magnetic resonance imaging was found to be 8%.^16^ Factors that are associated with the progression of a rotator cuff tear are an age of more than sixty years, a full-thickness tear, and fatty infiltration.^7^^,^^12^^,^^16^^,^^22^

However, the true prevalence of PTRCTs may be underreported. Of 249 cadaveric supraspinatus tendons examined, 13% had PTRCTs, of which 55% were intratendinous, 27% were articular surface, and 18% were bursal surface, suggesting that the vast majority of these intratendinous tears may have been overlooked in prior studies, due to the difficulties in identifying intratendinous tears with diagnostic imaging.^11^^,^^20^

Most of the current literature is directed toward the management of full-thickness rotator cuff tears. Current management of PTRCTs often presents a challenging situation for clinicians as there is no consensus on the optimal treatment. Current modes of treatment for PRCT involve nonoperative and operative management.^1^ Nonoperative interventions include physical therapy, nonsteroidal anti-inflammatory drugs, subacromial corticosteroid injections, and prolotherapy.^17^ These nonoperative care options are unable to specifically target the degenerative pathology of PRCT, especially within the hypovascularized tendon microenvironment.^6^ As biological treatment modalities continue to develop, platelet-rich-plasma (PRP) has shown promising effectiveness on the modulation of the inflammatory cascade and regeneration of functional tissue.^22^

There are three areas where PRP can be injected for PRCTs, which are yet to be standardized: intra-articular, subacromial, and intratendinous.^9^^,^^11^^,^^13^^,^^19^, ^20^, ^21^^,^^23^ Therefore, this paper will focus on reviewing the various injection sites of PRP in patients seeking nonsurgical PRCT with the intention of contributing to the standardization and optimization of PRP. This will be evaluated by reviewing current literature on functional outcome measures of the shoulder.

## Materials and methods

A literature search of two major electronic databases PubMed and Google Scholar was utilized. The search included randomized control trials (RCT) conducted between January 2010 and September 2021. Keywords include PRP, PTRCTs, intra-articular injection, subacromial injection, and intratendinous injection. Articles were then reviewed for adequacy according to the inclusion criteria: PTRCTs only, functional outcome scores pre-injection and post-injection, minimum 2-month follow-up time, and nonsurgical PRP injections only.

## Results

A total of eight RCT papers were obtained in the initial search and eight met the criteria for a total of 516 patients. Reported outcomes included DASH score (Disabilities of the Arm, Shoulder and Hand - one), Constant score (two), ASES score (American Shoulder and Elbow Surgeons - three), SST (Simple Shoulder Test - one), SDQ (Shoulder Disability Questionnaire - one), UCLA Shoulder Score (one), WORC (Western Ontario Rotator Cuff Index - two), SPADI (Shoulder Pain and Disability Index - two), and OSS (Oxford Shoulder Score - one).

There were no studies on leukocyte rich PRP (LR-PRP) and there were two studies on leukocyte poor PRP (LP-PRP), which contained 130 patients (Table I). The remaining six studies did not specify LR-PRP or LP-PRP. Follow-up duration for each study post PRP injection had an average of 7.1 months (Table II). Mean age of patients ranged from 39 to 59 years old across all included studies (Table II). In the intra-articular group, 30 patients in total received PRP. In the subacromial group, 148 patients in total received PRP. In the intratendinous group, 31 patients in total received PRP. Forty seven patients received combined subacromial bursa and intratendinous PRP (Table I).

**Table I:** Characteristics of selected partial rotator cuff tear studies.

Author (Year)	Type of study	Level of evidence	Intervention/control sample size	Location of injection	US guided vs. palpation guided	Frequency of injection	Platelet rich plasma volume	Platelet concentration
Ilhanli et al (2015)^10^	RCT	Level I	Interventions: PRP (n = 30) Control: PT (n = 32)	IA	NR	Weekly for 3 weeks	PRP (6 mL)	2.1-2.5x
Cai et al (2019)^3^	RCT	Level I	Interventions: SH + PRP (n = 48) SH (n = 44) PRP (n = 45) Control: NS (n = 47)	SA	US	Weekly for 4 weeks	PRP (4 mL)	NR
Kwong et al (2021)^13^	RCT	Level I	Intervention: LP-PRP (n = 47) Control: Corticosteroid (n = 52)	IT + SA	US	One injection	LP-PRP (3-5 mL)	1.6x
Kesikburn et al (2013)^11^	RCT	Level I	Interventions: PRP (n = 20) Control: NS (n = 20)	SA	US	One injection	PRP (5 mL)	NR
Thepsoparn et al (2021)^26^	RCT	Level I	Interventions: LP-PRP (n = 15) Control: Corticosteroid (n = 16)	LP-PRP injected into IT Corticosteroid injected into SA	US	One injection	LP-PRP (5 mL)	NR
Rha et al (2012)^20^	RCT	Level II	Interventions: PRP (n = 16) Control: Dry needling (n = 14)	IT	US	Two injections 4 weeks apart	PRP (2 × 3 mL)	NR
Ibrahim et al (2018)^9^	RCT	Level II	Interventions: PRP (n = 15) Control: Corticosteroid (n = 15)	SA	US	One injection	PRP (2 mL)	NR
Shams et al (2016)^24^	RCT	Level II	Interventions: PRP (n = 20) Control: Corticosteroid (n = 20)	SA	NR	One injection	PRP (2-2.5 mL)	NR

*RCT*, randomized control trial; *PRP*, platelet-rich plasma; *PT*, physical therapy; *SH*, sodium hyaluronate; *NS*, normal saline; *LP-PRP*, leukocyte poor PRP; *IA*, intra-articular; *SA*, subacromial; *NR*, not reported; *US*, ultrasound.

**Table II:** Partial rotator cuff studies with demographics and functional outcomes.

Author (Year)	Age range (mean +/− SD in y)	Follow-up interval	Functional outcome	Overall improvement	Baseline PROM	3 Month PROM and *P* value	6 Month PROM and *P* value	12 Month PROM and *P* value
Ilhanli et al (2015)^10^	Female: 57.62 ± 10.89 Male: 64.23 ± 6.29	12 months	DASH Work model	Both groups showed significant improvement in DASH and Work model DASH at 12-mo follow-up when compared to baseline (*P* < .05)	PRP: DASH score 66.26 ± 10.89 PT: DASH score 58.88 ± 14.07 PRP: DASH Work score 69.66 ± 19.02 PT: DASH Work score 56.25 ± 17.55	NA	NA	PRP: DASH score 35.62 ± 9.68 (*P* < .05) PT: DASH score 36.46 ± 14.58 (*P* < .05) *P* = .790 when comparing PRP DASH vs PT DASH PRP: DASH Work score 48.83 ± 77.40 (*P* < .05) PT: DASH Work score 30.78 ± 13.20 (*P* < .05) *P* = .199 when comparing PRP DASH Work vs PT DASH Work at 12 mo
Cai et al (2019)^3^	NS: 39.87 ± 8.96 SH: 38.93 ± 7.35 PRP: 40.56 ± 7.85 SH + PRP: 39.63 ± 7.65	3 mo 6 mo 12 mos 1 y MRI follow-up	Constant ASES	PRP and SH + PRP group showed significant increase in Constant and ASES score at all time points (*P* < .01)	NS Constant score: 56.51 ± 6.66 SH Constant score: 56.73 ± 3.72 PRP Constant score: 57.16 ± 2.47 SH + PRP Constant score: 55.94 ± 2.33 NS ASES: 49.19 ± 2.84 SH ASES: 49.59 ± 3.91 PRP ASES: 48.78 ± 3.32 SH + PRP ASES: 48.56 ± 3.58	NS Constant score: 58.11 ± 3.25 SH Constant score: 72.25 ± 2.56 (*P* < .05) PRP Constant score: 70.04 ± 3.18 (*P* < .05) SH + PRP Constant score: 77.56 ± 2.79 (*P* < .05) NS ASES: 48.72 ± 2.04 SH ASES: 61.95 ± 4.48 (*P* < .05) PRP ASES: 57.51 ± 5.49 (*P* < .05) SH + PRP ASES: 71.46 ± 4.2 (*P* < .05)	NS Constant score: 57.57 ± 2.69 SH Constant score: 69.55 ± 2.5 (*P* < .05) PRP Constant score: 76.73 ± 3.17 (*P* < .05) SH + PRP Constant score: 85.42 ± 2.05 (*P* < .05) NS ASES: 48.21 ± 2.52 SH ASES: 60.73 ± 5.67 (*P* < .05) PRP ASES: 67.89 ± 5.76 (*P* < .05) SH + PRP ASES: 80.92 ± 3.02 (*P* < .05)	NS Constant score: 56.49 ± 2.59 SH Constant score: 69.66 ± 2.43 (*P* < .05) PRP Constant score: 80.89 ± 2.55 (*P* < .05) SH + PRP Constant Score: 89.13 ± 2.26 (*P* < .05) NS ASES: 47.89 ± 2.56 SH ASES: 60.93 ± 4.65 (*P* < .05) PRP ASES: 75.8 ± 5.5 (*P* < .05) SH + PRP ASES: 89.38 ± 3.45 (*P* < .05)
Shams et al (2016)^24^	Corticosteroid: 50 ± 10 PRP: 52 ± 12	3 mo 6 mo MRI 6 mo follow-up	SST ASES CMS	SST, ASES, and CMS all showed significant improvement at 3 mo and 6-mo follow-up when compared to baseline (*P* < .01)	PRP SST: 6.3 ± 3 CS SST: 5.6 ± 3.1 PRP ASES: 52.6 ± 16 CS ASES: 52.5 ± 15 PRP CMS: 66 ± 21 CS CMS: 69.7 ± 19.4	PRP SST: 10.2 ± 1.8 (*P* < .01) CS SST: 8.2 ± 2.9 (*P* < .01) *P* = .013 when comparing PRP SST vs CS SST at 3 mo PRP ASES: 86.6 ± 12.2 (*P* < .01) CS ASES: 68.7 ± 12.3 (*P* < .01) *P* < .001 when comparing PRP ASES vs CS ASES at 3 mo PRP CMS: 90.9 ± 8.1 (*P* < .01) CS CMS: 77.4 ± 15.3 (*P* < .01) *P* = .001 when comparing PRP CMS vs CS CMS at 3 mo.	PRP SST: 10.2 ± 1.8 (*P* < .01) CS SST: 9.2 ± 2.7 (*P* < .01) *P* = .176 when comparing PRP SST vs CS SST at 6 mo PRP ASES: 83.4 ± 16.1 (*P* < .01) CS ASES: 78.9 ± 13.2 (*P* < .01) *P* = .340 when comparing PRP ASES vs CS ASES at 6 mo PRP CMS: 90.5 ± 8.3 (*P* < .01) CS CMS: 87.3 ± 12.2 (*P* < .01) *P* = .338 when comparing PRP CMS vs CS CMS at 6 mo.	NA
Ibrahim et al (2018)^9^	PRP: 46.8 ± 10.6 Corticosteroid: 41.5 ± 12.5	2 mo	SDQ	Significant improvement of 73% in SDQ score when compared to baseline (*P* < .001)	NA	NA	NA	NA
Kesikburn et al (2013)^11^	PRP: 45.5 ± 11.8 NS: 51.4 ± 10.0	3 weeks 6 weeks 3 mo 6 mo 1 y	WORC SPADI VAS+	WORC, VAS, and SPADI showed significant improvement at all time points for both control and intervention group when compared to baseline (*P* < .001)	PRP WORC: 34.6 (5.0-65.7) NS WORC: 29.9 (0.0-55.2) PRP SPADI: 77.5 (31.6-96.2) NS SPADI: 78.2 (33.6-100.0)	PRP WORC: 79.1 (19.8-96.6) *P* < .001 NS WORC: 58.5 (0.0-97.1) *P* < .001 PRP SPADI: 27.6 (1.2-84.0) *P* < .001 NS SPADI: 45.3 (2.9-95.5) *P* < .001	PRP WORC: 82.5 (16.9-100.0)*P* < .001 NS WORC: 69.9 (0.0-99.2) *P* < .001 PRP SPADI: 21.7 (0.0-96.1) *P* < .001 NS SPADI: 40.9 (0.0-95.5) *P* < .001	PRP WORC: 84.6 (26.7-100.0) *P* < .001 NS WORC: 79.7 (0.0-99.3) *P* < .001 PRP SPADI: 14.6 (0.0-86.3) *P* < .001 NS SPADI: 15.4 (0.0-96.0) *P* < .001
Thepsoparn et al (2021)^26^	PRP: 51.3 ± 10.3 Corticosteroid: 62.4 ± 10.5	1 mo 6 mo	OSS	In the PRP group, OSS had a statistically significant 53.8% improvement when compared to baseline (*P* < .01)	PRP OSS: 35.1 ± 7.6 CS OSS: 36.6 ± 7.2	NA	PRP OSS: 16.2 ± 3.9 (*P* < .01) CS OSS: 25.0 ± 10.2 (*P* < .01) *P* < .01 when comparing PRP OSS vs CS OSS at 6 mo.	NA
Rha et al (2012)^20^	PRP: 52.2 ± 9.5 Dry needling: 53.9 ± 11.6	2 weeks 4 weeks 6 weeks 3 mo 6 mo US evaluation 6 mo post injection	SPADI	Both groups showed statistically significant improvement in SPADI score with PRP showing improved clinical effects at earlier time points (*P* < .05)	PRP SPADI: 62.3 ± 4.1 Dry needling SPADI: 62.8 ± 4.2	PRP SPADI: 21.1 ± 3.9 (*P* < .05) Dry needling SPADI: 34.6 ± 4.0 (*P* < .05) *P* < .05 when comparing PRP SPADI vs Dry Needling SPADI at 3 mo.	PRP SPADI: 17.7 ± 3.7 (*P* < .05) Dry needling SPADI: 29.5 ± 3.8 (*P* < .05) *P* < .05 when comparing PRP SPADI vs Dry Needling SPADI at 6 mo.	NA
Kwong et al (2021)^13^	LP-PRP: 49.94 ± 9.70 CS: 49.08 ± 9.54	6 weeks 3 mo 12 mo	ASES WORC	LP-PRP group showed significantly increased ASES and WORC score at 3 and 12 mo follow-ups when compared to baseline (*P* < .02)	LP-PRP ASES: 53.9 ± 15.8 CS ASES: 61.8 ± 17.2 LP-PRP WORC: 42.2 ± 15.6 CS WORC: 49.5 ± 17.2	*Change in PROM (delta)* LP-PRP ASES: 13.0 ± 18.7 CS ASES: 2.9 ± 22.5 *P* = .02 when comparing PRP ASES vs CS ASES at 3 mo. LP-PRP WORC: 16.8 ± 19.0 CS WORC: 5.8 ± 25.1 *P* = .03 when comparing PRP WORC vs CS WORC at 3 mo.	NA	*Change in PROM (delta)* LP-PRP ASES: 19.2 ± 19.4 CS ASES: 11.9 ± 23.3 *P* = .15 when comparing PRP ASES vs CS ASES at 12 mo. LP-PRP WORC: 22.3 ± 25.2 CS WORC: 15.1 ± 21.8 *P* = .19 when comparing PRP WORC vs CS WORC at 12 mo.

*SD*, standard deviation; *PROM*, patient reported outcome measures; *SA*, subacromial; *IT*, intratendinous; *IA*, intra-articular; *NR*, not reported; *US*, ultrasound; *PT*, physical therapy; *SH*, sodium hyaluronate; *NS*, normal saline; *LP-PRP*, leukocyte poor PRP; *DASH*, Disabilities of Arm, Shoulder, and Hand; *ASES*, American Shoulder and Elbow Surgeons; *SST*, Simple Shoulder Test; *CMS*, Constant-Murley Score; *SDQ*, Shoulder Disability Questionnaire; *WORC*, Western Ontario Rotator Cuff; *SPADI*, Shoulder Pain and Disability Index; *VAS*, Visual Analog Scale; *OSS*, Oxford Shoulder Score.

In the included articles, whether PRP is leukocyte-rich or not is often related to whether it contains interlayer substances in the separation process and whether the concentration of leukocytes in PRP is higher than that in autologous blood. Of the studies, two involved LP-PRP, none involved LR-PRP, while six studies did not specify (Table I).

When looking at the breakdown by injection site, one paper utilized the intra-articular route, four studies used a subacromial approach, two studies used an intratendinous approach, and one combined a subacromial and intratendinous approach (Table I). A total of 256 patients received PRP injections across eight separate studies. One hundred forty eight patients had subacromial injections making up the largest group (58%), followed by 47 combined intratendinous and subacromial injections (18%), followed by 31 intratendinous injections (12%), and 30 intra-articular injections (12%). The only reported complication reported in the studies was temporary pain early after PRP treatment. No cases of fever, rash, swelling, hematoma, skin atrophy, loss of pigmentation, or infection were reported after PRP injection.

In order to analyze the results of the functional measures based on PRP location, the improvement was calculated as a percentage change from initial baseline scores to post-treatment scores. This was calculated for time periods of at least 2 months post-injection up to 12 months post-injection (Table II).

### Intra-articular injections

Based on the inclusion criteria, an RCT was found comparing intra-articular PRP injection with physical therapy (PT) in patients with chronic partial supraspinatus tears. Ilhanli et al randomized patients into two groups: PRP (n = 30) and PT (n = 32).^10^ The total number of platelets in the PRP per mL represented a mean increase of 2.1-2.5 times compared with whole blood values. Functional outcome measures of the shoulder included range of motion (ROM), Disabilities of the Arm, Shoulder, and Hand (DASH), and work model score of DASH. The intervention group consisted of three palpation-guided intra-articular 6 mL injections of PRP into the glenohumeral joint with one week interval between each injection, in comparison to 15 PT sessions (5 sessions per week for three weeks). Both groups improved from baseline in ROM, DASH score, and work model score of DASH. When compared to PT alone at the 12-month follow-up, intra-articular PRP did not show significant improvement in ROM.^10^ Interestingly, before the treatment, the DASH score and work model of DASH score were significantly higher in the PRP group compared to the PT group. However there was no difference between the PT and PRP treatment groups for these scores at the 12 month follow-up (*P* = .199).^10^ In looking at the overall percent improvement in the PRP vs PT group, both groups showed statistically significant increase (*P* < .05) at 12 months in the DASH and work model DASH. Where the PRP group had a 46.2% improvement in DASH score and a 29.9% improvement in work model DASH score at 12-month follow-up, the PT group improved by 38% at the 12-month follow-up and the work model DASH improved by 45.3%.

### Subacromial injection

Four studies utilized the subacromial route of injection.^3^^,^^9^^,^^11^^,^^24^ In a double-blind RCT conducted by Cai et al, patients were divided into four groups: normal saline (NS), n = 47, sodium hyaluronate (SH) group, n = 44, PRP group, n = 45, and a PRP + SH group, n = 48, and evaluated their efficacies in treating supraspinatus PTRCT.^3^ Key functional outcome measures were measured to look at treating bursal-sided PTRCT including the Constant and American Shoulder and Elbow Surgeons (ASES) scores at 1, 3, 6, and 12-months post-treatment (1-month follow-up excluded for our review). Using an ultrasound-guided injection technique, there were two groups with PRP divided into a PRP (4 mL) and PRP (2 mL) combined with SH (2 mL) into the PRP + SH group. The NS and SH group each received 4 mL of their respective formulations. These subjects received injections weekly for four consecutive weeks. All four groups showed improvements in both Constant and ASES scores when comparing pre-injection to post-injection except for the NS group from baseline. The SH group showed statistical significance when comparing ASES at 3, 6, and 12 months to 1 month scores. But between the four groups, only PRP and SH + PRP were the groups which showed statistical differences (*P* < .05) in both Constant and ASES when comparing 12 months to 6 months scores.^3^ Overall, there was no statistical difference between the PRP and SH + PRP group.

Shams et al conducted an RCT with 40 patients having MRI evidence of a partial supraspinatus tear who were treated with either a subacromial corticosteroid injection or a single injection of 2 to 2.5 mL PRP.^24^ A palpation guided approach was used for both patient groups. There was statistical improvement across all follow-up time-points in both groups on ASES, CMS, and SST scores. Interestingly, those who received PRP had statistically significant difference compared to corticosteroid at 3 months in ASES, CMS, and SST scores but showed no statistically significant difference at 6 months follow-up between the groups.^24^

Ibrahim et al compared the efficacy of ultrasound (US)-guided PRP vs. corticosteroid injection for treatment of rotator cuff tendinopathy (RCT).^9^ 30 patients with RCT were randomly divided into two groups of fifteen patients. One group received a single US-guided injection of PRP (2 mL) using a subacromial lateral approach and the other group received US-guided corticosteroid injection. These patients were then evaluated using Shoulder Disability Questionairre (SDQ) at baseline and 2 months. Compared to baseline, both corticosteroid and PRP injections significantly improved in SDQ measures (*P* < .01). However, there were no significant differences in SDQ at 2 months follow-up between the groups.

Kesikburun et al conducted a randomized double-blind placebo-controlled trial including 40 patients with chronic rotator cuff tendinopathy where they were randomly assigned to receive either a 5mL PRP injection (n = 20) or 5mL saline injection (n = 20) under ultrasound guidance into the subacromial bursa.^11^ WORC and SPADI were compared at 3 months, 6 months, and 12 months after injection. When assessing the PRP vs saline, WORC and SPADI scores at all time points showed statistically significant improvement when compared to baseline (*P* < .001).^11^

### Intratendinous injection

Within the category of intratendinous PRP injection studies, three studies were found that had follow-up period of at least 3 months. Kwong et al performed a randomized control trial comparing corticosteroid injection with intratendinous LP-PRP injection in 99 patients. At 3 months post-injection, the PRP group had significant functional outcome improvement compared to corticosteroid injection in both the Western Ontario Rotator Cuff Index (WORC) and ASES.^13^ At 3-month follow-up, PRP injections showed a significant improvement in the ASES when compared to the corticosteroid group (*P* = .02) and no significant changes in ASES at the 12-month follow-up (*P* = .15).^13^ At the 3-month follow-up, PRP injection also showed a significant improvement in the WORC (*P* = .03) but no significant changes at the 12-month follow-up (*P* = .19).^13^

Rha et al performed two PRP (3 mL) injections directly into the supraspinatus tendon under ultrasound guidance 4 weeks apart vs dry needling.^20^ PRP was found to be superior in SPADI scores at 66.1% improvement (*P* < .05) at 3 months and 71.6% at 6 months (*P* < .05) after two PRP injections.^20^

Thepsoparn et al compared one US-guided LP-PRP (n = 15) vs. corticosteroid injection (n = 16). The LP-PRP was injected intra-tendinous, while corticosteroids were injected into the subacromial bursa.^26^ At the 6-month follow-up, the PRP group showed significant improvement in OSS compared to the corticosteroid group 53.8% vs 31.7% (*P* < .01).

## Discussion

The most important finding of this literature review is that regardless of injection site location, all PRP injections had notable functional improvement in PRCTs.^3^^,^^9^, ^10^, ^11^^,^^13^^,^^19^, ^20^, ^21^^,^^23^^,^^24^ The results continued to show benefits and functional outcome improvements at various time points from as early as 3 months post-injection through 12 months.^3^^,^^9^, ^10^, ^11^^,^^13^^,^^19^, ^20^, ^21^^,^^23^^,^^24^ However, the differing functional outcome scores used for follow-up, variability in the type of PRP used, and injection locations caused difficulty in comparing the studies. It has been shown that some of the functional outcome measures used in the studies have a high correlation with one another and thus can be compared in the shoulder. These functional outcome measures include: ASES, SPADI, and QDASH/DASH.^5^ Consequently, we have focused our comparative discussion on studies that include these functional outcome measures and overlap in time period (3 months, 6 months, and 12 months post injection).

With only a single study utilizing PRP intra-articularly, there was statistically significance of improvement in DASH at 12 months in both the PT and PRP group. This is not enough data to suggest whether consideration of the intra-articularly joint space should be considered when administering PRP in PRCTs. A severe limitation of the current literature available is not clarifying if ultrasound guidance was utilized. A study by Borbas et al compared cadaveric injection accuracy of palpation guided glenohumeral joint injections showing 89.6% with posterior, 75% anterior, and 54.2% superior with both posterior and anterior are statistically significantly more accurate than superior injections.^2^ While another study showed 96.6% accuracy using ultrasound guidance for glenohumeral joint injections.^18^ Depending on the approach utilized for the study, it could have had inaccurate location of where PRP was injected. Thus, it is not enough to make a conclusion if intra-articular joint should be recommended as part of the administration of PRP with PRCTs. Further studies need to better design with the utilization of ultrasound guidance in the administration of glenohumeral joint injections.

Shams et al showed that subacromial PRP injections showed better results at the 3-month timeframe when compared to subacromial corticosteroid injections in the ASES, CMS, SST, and VAS scores. ^24^ However, both PRP and corticosteroids showed similar outcomes at the 6-month follow-up for ASES, CMS, and SST scores.^24^ MRI, however, noted nonsignificant improvements in the grade of the tendinopathy and MRI improvements between both groups.^24^ Another consideration is the utilization of MRI to measure outcomes of PRP treatment may not be as efficacious as ultrasound imaging which needs to be further evaluated.

Cai et al demonstrated the efficacy of subacromial PRP and PRP + SH injections, which both showed significant increases in Constant and ASES scores at the 3-month, 6-month, and 12-month time periods.^3^ On the other hand, NS injections did not show statistical significance at any time frame. Interestingly, SH injections alone did show improvement at the follow-up periods. However, the improvements were not as markedly impressive as in combination with PRP.

Three studies compared PRP to corticosteroid. Thespsoparn et al showed LP-PRP intratendinous injection having a statistical significance at 6 months in OSS when compared to subacromial bursa corticosteroid injection.^26^ Despite this conclusion, the PRP group had statistically significantly younger patient population than the corticosteroid group (51.3 years vs 62.4 years, *P* = .006), which could be a potential confounder in the results when comparing the efficacy of PRP and corticosteroids. Kwong et al showed a combined intratendinous and subacromial bursa LP-PRP group showed significantly increased ASES and WORC score at 3 and 12 month follow-ups when compared to baseline, however when compared to corticosteroid, PRP only had significant difference at the 3-month timeframe and not at 12 months. Ibrahim et al had an ultrasound guided PRP vs corticosteroid into the subacromial bursa. Their findings revealed that either injection method showed marked improvement at the 2-month follow-up in the SDQ score and ROM maneuvers including flexion, abduction, extension, internal, and external rotation.^9^ However, there were no significant differences when comparing the two groups. Notably, post-treatment ultrasound imaging showed slightly greater improvements in the outcomes of partial tears and effusions in the PRP group vs. the corticosteroid group.

When looking at the studies analyzed, there was no mention of articular or bursal sided tearing which could provide better direction in respect to how each type of PRCT responds to PRP. Additionally, not all of the studies had a consistent definition or measurement of what was considered partial rotator cuff tear which presents difficulty when trying to compare the various tendons across studies. A weakness of the review is the heterogeneity of tendon architecture within each study. There was not a clear delineation between underlying tendinosis and concurrent PRCT in a majority of the studies listed. This presents a challenge when attempting to interpret the current data as tendinosis could respond differently to PRP than partial tears. Only Kesikburn et al explicitly describes tendinosis in their population (shown in Tables III and IV) and although both the saline and PRP group had statistically significant differences at all time points this may indicate that tendinosis in isolation may be more responsive to PRP rather than if there is a concurrent partial tear present.

**Table III:** Characteristics of selected rotator cuff tendinosis studies.

Author (Year)	Type of study	Level of evidence	Intervention/control sample size	Location of injection	US guided vs. palpation guided	Frequency of injection	Platelet rich plasma volume	Platelet concentration
Kesikburn et al (2013)^11^	RCT	Level I	Interventions: PRP (n = 20) Control: NS (n = 20)	SA	US	One injection	PRP (5 mL)	NR

*RCT*, randomized controlled trial; *SA*, subacromial; *US*, ultrasound; *PRP*, platelet-rich plasma; *NR*, not reported.

**Table IV:** Rotator cuff tendinosis studies with demographics and functional outcomes.

Author (Year)	Age range (mean +/− SD in y)	Follow-up interval	Functional outcome	Overall improvement
Kesikburn et al (2013)^11^	PRP: 45.5 ± 11.8 NS: 51.4 ± 10.0	3 weeks 6 weeks 3 mo 6 mo 1 y	WORC SPADI VAS+	WORC, VAS, and SPADI showed significant improvement at all time points for both control and intervention group when compared to baseline (*P* < .001)

*SD*, standard deviation; *WORC*, Western Ontario Rotator Cuff; *SPADI* Shoulder Pain and Disability Index; *VAS*, visual analog scale; *NS*, normal saline; *PRP*, platelet-rich plasma.

Another limitation of the review was the lack of standardization using imaging guidance to inject in the subacromial bursa, intratendinous, and into the glenohumeral joint. Without confirmation of image guidance during the intervention, the injectate could have been placed in a suboptimal location. Furthermore, some clinicians provide needle fenestration in conjunction with PRP administration which was not reported consistently throughout the studies. Finally, the differences in the PRP preparation were not stated consistently throughout the studies, and only two studies performed analysis of the concentration of final PRP obtained. Ideally to further clarify where the optimal location of PRP should be injected, the concentration and total number of platelets should be analyzed to not have variation across the differences among what is being injected in determining whether LR or LP have a superior response.

Based on our review of current literature involving RCT studies of PRP therapy, there is inconclusive evidence of where the ideal location is to inject PRP when PRCT is present. Additional higher quality studies are needed to elucidate which is the optimal location.

## Conclusion

The use of PRP in orthopedic disorders has been an increasingly emerging treatment option. While PRP shows exciting promise, there has yet to be a consensus on how PRP is best utilized in treating the rotator cuff. There are several weaknesses in the current body of available literature in existence and proper comparisons cannot be made due to a high level of heterogeneity across these studies. Consequently, there are several limitations that should be leveraged from existing publications which should be considered for the future standard of research.

The quality of PRP injectate was not analyzed, which leaves ambiguity as to what type of PRP (LR or LP) patients received. Without distinguishing between the composition of the platelets, it poses difficulty in what exactly was injected. There could have been a wide variation in the number of red blood cells and leukocytes between groups which may yield to different outcomes. With regards to the number of total injections in the PRP groups varies from one to four, making it difficult to find the optimal number of PRP injections for PRCT. There is variation in the time interval between injections and it should be standardized in future studies. Something lacking throughout the current body of literature is a consensus on the post procedural instructions in which is followed by patients and physical therapist to guide post procedural treatment plans.

Finally, some of the studies used ultrasound guidance for injections while others performed palpation guided procedure. Our review displays the shortcomings and the lack of definitive conclusions that can be made from current literature to utilize PRP in PRCTs as an effective treatment option. Future double-blind, randomized, controlled studies are warranted to support further explore the utility of PRP in PRCTs. Another direction would be the use of precision medicine in determining which tendon or if multiple tendons are affected and personalizing the injection location for that individual. Because more literature exists on supraspinatus PRP injections, it would be beneficial to see the effect of PRP on other rotator cuff tendons as well. Overall, more standardized research is needed to further optimize who is the optimal candidate, concentration of platelets, dose of platelets, and procedural technique when utilizing PRP for PRCT.

## Acknowledgments

The authors thank Elizabeth Sosic, MSL who assisted in management, preparation, and submission of this manuscript for publication.

## Disclaimers:

Funding: No funding was disclosed by the authors.

Conflicts of interest: The authors, their immediate families, and any research foundation with which they are affiliated have not received any financial payments or other benefits from any commercial entity related to the subject of this article.
